# Supplementary material for: Sturgeon Chondroitin Sulfate Restores the Balance of Gut Microbiota in Colorectal Cancer Bearing Mice
Source: Int J Mol Sci. 2022 Mar 28;23(7):3723. doi: 10.3390/ijms23073723 (PMC9040715; doi:10.3390/ijms23073723)
Supplement: Supplementary file 1 [file ijms-23-03723-s001.zip › ijms-1592249-supplementary (1).pdf]

Supplementary data

Sturgeon chondroitin sulfate restores the balance of gut micro-biota in colorectal cancer bearing mice

Ruiyun Wu[1,2], Qian Shen[3], Pinglan Li[1*], Nan Shang[2,4*]

1 Key Laboratory of Precision Nutrition and Food Quality, College of Food Science and Nutritional Engineering, China Agricultural University, Beijing 100083, China; wry0814@cau.edu.cn

2 College of Engineering, China Agricultural University, Beijing 100083, China

3 Department of Biology, Rhodes College, 2000 North Pkwy, Memphis, TN 38112, USA; shenq@rhodes.edu

4 Key Laboratory of Precision Nutrition and Food Quality, Department of Nutrition and Health, China Agricultural University, Beijing 100083, China

* Correspondence: lipinglan@cau.edu.cn (P.L.); nshang@cau.edu.cn (N.S.);

Tel./Fax: +86-010-6273-8678 (P.L.)

Aa

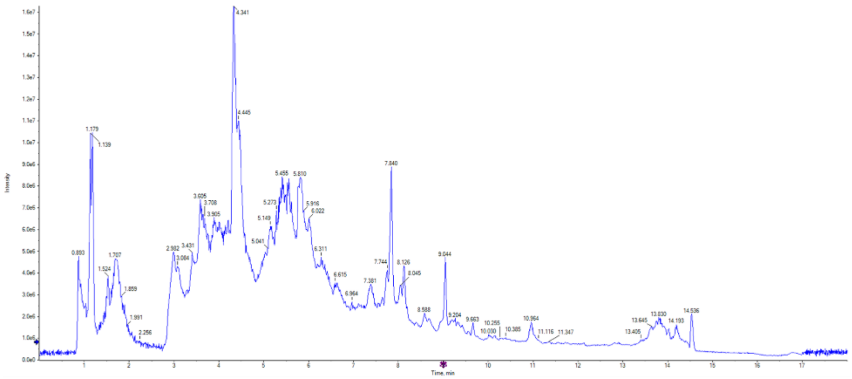

Ab

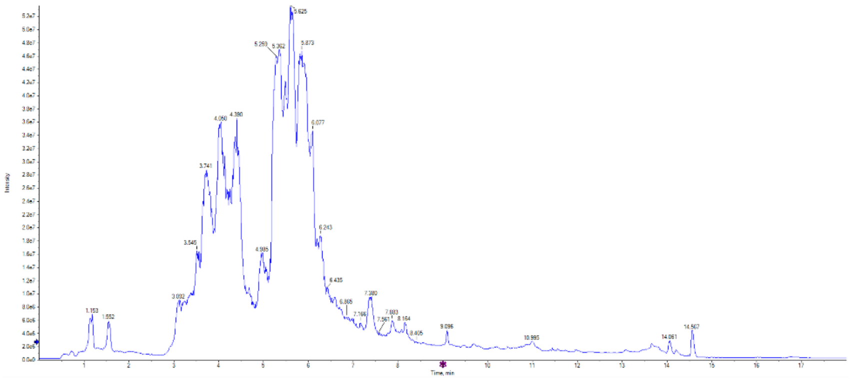

Ba

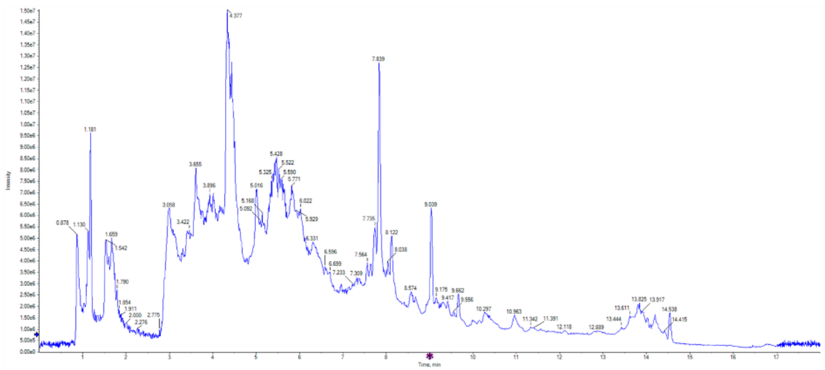

Bb

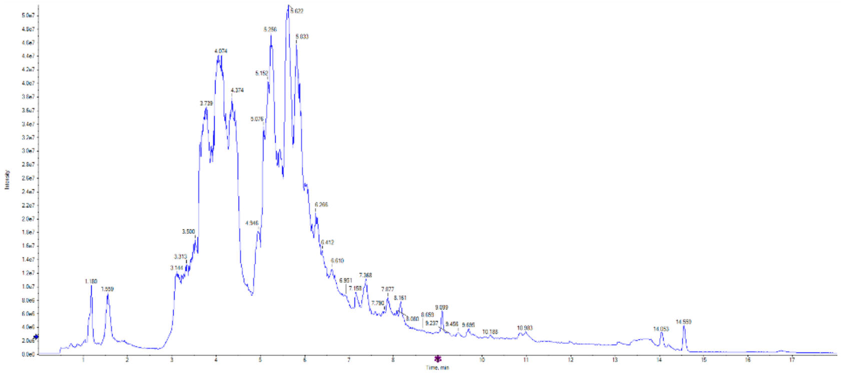

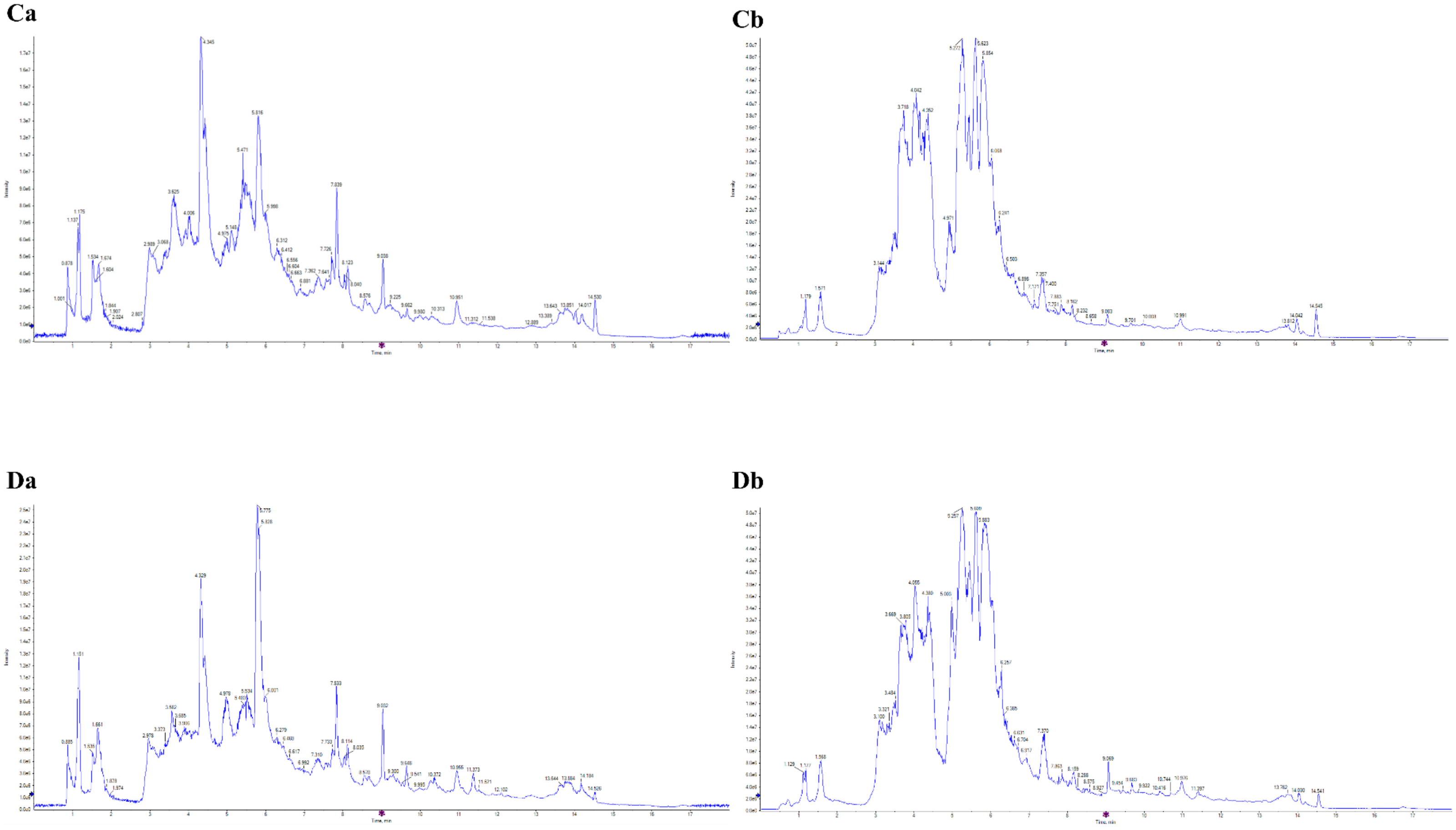

Figure S1. The representative UHPLC-QTOF/MS total ion chromatogram (TIC) of urine in Model group (A), Model group (B), DDP group (C) and High group (D) were analyzed in positive (a) and negative (b) mode respectively.
